# The Differential Antagonistic Ability of Curcumin against Cytotoxicity and Genotoxicity Induced by Distinct Heavy Metals

**DOI:** 10.3390/toxics11030233

**Published:** 2023-02-28

**Authors:** Qiao Liu, Mengzi Sun, Tong Wang, Yemian Zhou, Meng Sun, Han Li, Yun Liu, An Xu

**Affiliations:** 1 School of Basic Medical Sciences, Anhui Medical University, No. 81, Meishan Road, Hefei 230032, China; 2 Anhui Province Key Laboratory of Environmental Toxicology and Pollution Control Technology, High Magnetic Field Laboratory, HFIPS, Chinese Academy of Sciences, Hefei 230031, China

**Keywords:** curcumin, heavy metals, detoxification, bioaccumulation, anti-oxidation

## Abstract

Widespread heavy metal pollution has aroused severe health risks worldwide. Curcumin has been reported to play a wide-spectrum protective role for various heavy metals. However, the specificity and difference in the antagonistic ability of curcumin against distinct types of heavy metals are still largely unknown. Here, using cadmium (Cd), arsenic (As), lead (Pb), and nickel (Ni) as the typical heavy metals, we systematically compared the detoxification efficiency of curcumin on the cytotoxicity and genotoxicity elicited by different heavy metals under the same experimental conditions. Curcumin was proved to have a significant discrepant antagonistic capacity when counteracting the adverse effect of different heavy metals. Stronger protective effects of curcumin emerged when antagonizing the toxicity of Cd and As, rather than Pb and Ni. Curcumin exhibits a better detoxification ability against heavy metal-induced genotoxicity than cytotoxicity. Mechanistically, inhibiting the oxidative stress elicited by heavy metals and reducing the bioaccumulation of metal ions both contributed to the detoxification of curcumin against all the tested heavy metals. Our results illustrated that curcumin shows prominent detoxification specificity against different types of heavy metals and toxic endpoints, which provides a new clue for the better and targeted application of curcumin in heavy metal detoxification.

## 1. Introduction

Global industrialization and urbanization have inevitably aggravated the incidence of heavy metal pollution in atmospheres, soils, and water bodies for almost a century [1,2]. Consuming contaminated water and foods irrigated with polluted water has been the main source of human exposure to heavy metals [3]. The tailings samples of a large gold mine in South Korea were reported to contain a series of heavy metals; the concentrations of nickel, arsenic, cadmium, zinc, and lead all exceed the reference standards for soil pollution. Specifically, the average values of arsenic and cadmium were about 30 to 120 times higher than the standard [4]. Children living near the smelter or whose father was a smelter worker were 19.3 and 4.4 times more likely to have blood lead levels (BLLs) higher than 5 µg/dl, respectively [5]. Due to the properties of extreme toxicity, high capability of bioaccumulation, and low efficacy of biological excretion, heavy metal pollution is a severe health risk worldwide [6,7,8]. Increasing epidemiologic and laboratory research has associated environmental heavy metal exposure with injuries to the nervous, digestive, cardiovascular and reproductive systems [9,10,11]. More importantly, genotoxicity is a proven adverse effect elicited by heavy metals exposure. Cadmium (Cd), arsenic (As), and lead (Pb) all have been shown to cause several genetic abnormalities, such as micronucleus formation, chromosomal abnormalities, and DNA damage [12,13,14]. Since genotoxicity is the prerequisite for the incidence of most malignancies, a series of heavy metals have been classified as Group I carcinogenic agents by the International Agency for Rese [15]. Itai-itai disease, Minamata disease and cadmium-polluted rice caused by heavy metal contaminations have all seriously threatened human health throughout history [16,17].

Various technologies have been adopted to alleviate the increasingly serious pollution of heavy metals. At present, the main treatments for environmental heavy metals are chemical precipitation, electrolysis, ion exchange, membrane separation, and adsorption [18,19,20]. Anti-oxidation, anti-apoptosis, and metal chelation are common strategies for heavy metal detoxification in laboratory studies [21]. Only symptomatic treatments (i.e., emetic, gavage, catharsis, and pain relieving) and non-specific metal-chelators can be used to cure patients with heavy metal poisoning in current clinical treatment, which have limited therapeutic efficiency and may cause unexpected side effects [22]. Therefore, the development of effective and safe antidotes is urgently needed to protect humans from heavy metal pollution.

Curcumin is a hydrophobic polyphenol natural compound mostly concentrated in the rhizome of Curcuma longa [23]. In addition to being a popular dietary spice around the world, curcumin also exhibits various beneficial biological activities including being anti-oxidant, anti-inflammatory, anti-tumor, and anti-bacterial [24]. A series of authoritative international institutions, such as the Food and Drug Administration (FDA) in the USA and the Joint FAO/WHO Expert Committee on Food Additives, have confirmed the safety of curcumin in daily use and clinical treatment [25,26]. Depending on the outstanding biological properties, curcumin is increasingly employed to antagonize heavy metals’ adverse effects, mainly through anti-oxidation and metal chelation [27,28]. For instance, curcumin had been shown to have many therapeutic properties on cadmium-induced organ toxicity by removing ROS and inhibiting oxidative damage [29]. In one set of in vivo experiments, it was found that exposure to lead and cadmium increased hypertension and oxidative stress in mice, and curcumin effectively ameliorated these adverse events in metal-exposed animals [30]. In addition, Kabeer Abubakar et al. showed that curcumin attenuates Pb-induced neurotoxicity by inhibiting oxidative stress and chelating Pb ions [31].

Although numerous research has reported the protective role of curcumin against heavy metals, most previous research focused on investigating the detoxification ability of curcumin against a single type of heavy metal, therefore, it was difficult to compare the efficacy and specificity of curcumin against diverse heavy metals due to the inconsistent antagonistic results originating from the distinct experimental systems and evaluation criteria. For example, Somparn et al. reported that curcumin inhibits Cd-induced cytotoxicity by about 80%; Cd-induced cell survival proportion was elevated from about 50% to approximately 90% after curcumin treatment [32]. However, in another similar study, curcumin was demonstrated to decrease the toxicity of Cd only by about 29% [33]. Similar inconsistent conclusions also existed for the combined effects of curcumin and other heavy metals. Negligible protective effects of curcumin could be observed when combating the cytotoxicity elicited by As in embryonic fibroblast cells [34], however, curcumin was proved to decline the As-induced mortality rate by 40% in PC12 cells [35]. As a promising antidote with high efficiency and excellent biocompatibility, it is of great value to systematically evaluate the detoxification effect of curcumin against different typical heavy metals under the same experimental standards.

Cd, As, Pb, and nickel (Ni), which are the main inorganic pollutants widely existing in the natural environment [36], were selected as typical types of heavy metal in this study. Using HepG2 cells originating from the liver, a main target organ for heavy metal poisoning [37], our objective was to systematically compare the protective role of curcumin on different heavy metals, and identify the specificity and high efficacy of curcumin in antagonizing certain types of heavy metals, to better understand the application of curcumin in heavy metal detoxification. Our data illustrated that a significant difference in antagonistic efficiency exists when treating various heavy metals with curcumin. The protective role of curcumin on genotoxicity elicited by heavy metals was more obvious than cytotoxicity mainly through anti-oxidation and reducing the bioaccumulation of heavy metals. This study provides novel information for understanding and guiding the application of curcumin in distinct types of heavy metal detoxification. 

## 2. Materials and Methods

### 2.1. Chemical Reagents

Cadmium chloride (CdCl_2_·2.5H_2_O, ≥99% purity), sodium arsenite (NaAsO_2_, ≥99.5% purity), nickel chloride hexahydrate (NiCl_2_·6H_2_O, ≥98% purity), and lead acetate ((CH3COO)_2_Pb, ≥99% purity) were obtained from Sinopharm Chemical Reagent Co., Ltd. (Shanghai, China). The above chemicals are referred to simply as Cd, As, Ni and Pb hereafter. Curcumin was obtained from Sigma-Aldrich (USA). Cell counting kit-8 (CCK-8) was purchased from Biosharp (Guangzhou, China). A lactate dehydrogenase (LDH) detection kit was purchased from Nanjing Jiancheng Bioengineering Institute (Nanjing, China). Annexin V-FITC/PI Apoptosis Detection Kit was purchased from BD Pharmingen (Franklin Lake, NJ, USA). Phosphorylated histone H2AX (γ-H2AX) antibody was obtained from Novus Biologicals (Littleton, CO, USA). Horseradish peroxidase (HRP) conjugated anti-mouse antibodies were bought from Proteintech (Chicago, IL, USA).

### 2.2. Cell Culture and Co-Exposure Manner

A human hepatic carcinoma (HepG2) cell line was purchased from the Shanghai Cell Bank of Type Culture Collection of China. Cells were cultured continuously at 37 °C and 5% CO_2_ in Dulbecco’s modified Eagle medium (DMEM) medium (Cytiva, Results Way, Marlborough, MA, USA) with 10% fetal bovine serum (FBS) and 100 units/mL penicillin/streptomycin (Invitrogen, Carlsbad, CA, USA). To verify the effect of curcumin on the detoxification effects of different heavy metals, we treated curcumin at non-toxic concentrations (5, 10 and 20 µM). The stock solution of curcumin and heavy metals were diluted with DMEM to the concentration of the working solution and then exposed to cells.

### 2.3. Cell Viability Assessment

To examine the individual toxicity of curcumin and heavy metals as well as their combined toxicity, cells were first seeded in 96-well plates at a density of 1.5 × 10^4^ cells per well for 24 h. Cells were then exposed to curcumin or different heavy metals for an indicated time. After incubation, the cell culture medium was removed, and the cells were washed thrice with PBS. After that, CCK-8 with a final concentration of 10% (*v*/*v*) was added to each well for 2–3 h at 37 °C. The absorbance was recorded on a spectra Max i3x microplate reader (Molecular Devices, Sunnyvale, Silicon Valley, CA, USA) at 450 nm.

### 2.4. Lactate Dehydrogenase (LDH) Detection

Cells were plated in 6-well culture plates at a density of 6 × 10^5^ cells per well for 24 h. After treating cells with curcumin or heavy metals, the culture supernatant of each well in the 6-well plate was collected and centrifuged for further detection using a commercial LDH Kit, according to the manufacturer’s instructions. The absorbance was detected at 450 nm by a spectra Max i3x microplate reader (Molecular Devices, Sunnyvale, Silicon Valley, CA, USA).

### 2.5. Apoptosis Assay

Cells were plated in 6-well culture plates at a density of 6 × 10^5^/well. Cells were pretreated with curcumin for 8 h and then incubated with heavy metals for an additional 24 h. The cells were then trypsinized, collected, and washed twice with ice-cold PBS. The apoptosis rates of HepG2 cells were detected by Annexin V-FITC/PI detection kit. The collected cells were re-suspended in 300 μL binding buffer containing 5 μL Annexin V-FITC stock solution and 5 μL PI stock solution and incubated in dark at room temperature for 15 min. Cellular apoptosis was immediately analyzed by flow cytometry (BD Biosciences, Franklin Lake, NJ, USA). Approximately 2 × 10^4^ cells were analyzed in each sample.

### 2.6. Western Blotting Analysis

Cells treated with curcumin and heavy metals were collected and lysed in a radioimmunoprecipitation assay (RIPA) lysis buffer (Beyotime, Shanghai, China) on ice. Proteins extracted from the cells were separated by 15% sodium dodecyl sulfate polyacrylamide gel electrophoresis (SDS-PAGE) and transferred onto polyvinylidene fluoride (PVDF) membranes (Roche, Basel, Swiss). The PVDF membrane was blocked with Tris-Buffered Saline Tween-20 (TBST, 0.1% Tween-20) which contained 5% nonfat dry milk at room temperature for 1 h, and then incubated with appropriate dilutions of γ-H2AX primary antibody at 4 °C overnight. After washing with TBST six times, the PVDF membrane was then incubated with HRP-conjugated secondary antibodies (1:10000) for 1 h at room temperature. The autoradiographic signals were monitored on a Tanon-5200 Chemiluminescence Imaging System (Tanon, Shanghai, China). *β*-Actin was used as the internal control in the experiments.

### 2.7. ROS Detection

Cells were seeded in 96-well plates and cultured for 24 h. Curcumin and heavy metals were added into the cells and incubated for another 24 h. After the treatments, cells were washed with PBS and incubated with 2′,7′-dichlorofluorescin diacetate (DCFH-DA, ROS detection probe) and Hoechst 33342 (a kind of commonly used probe for cellular nuclear staining) for 30 min at 37 °C in the dark. DCFH-DA (λ_Excitation_ = 488 nm, λ_Emission_ = 525 nm) and Hoechst 33342 fluorescence (λ_Excitation_ = 352 nm, λ_Emission_ = 461 nm) were recorded separately using a spectra Max i3x microplate reader (Molecular Devices, Sunnyvale, Silicon Valley, USA). The content of ROS production was calculated by the fluorescence ratio of DCFH-DA to Hoechst 33342.

### 2.8. Quantitatively Detection of the Bioaccumulation of Various Heavy Metals

For quantitative analysis of the cellular accumulation of heavy metals, cells cultured in a 60 mm dish were treated with different heavy metals with or without curcumin co-treatment. After incubation, cells were collected, washed with PBS, and then nitrified with HNO_3_ at 70 °C for 72 h. The intracellular bioaccumulation of Cd, Pb, and Ni was measured using inductively coupled plasma mass spectrometry (ICP-MS, Thermo Fisher Scientific, Waltham, MA, USA). The cellular accumulation of As was assessed by an atomic fluorescence spectrometer (AFS, Jitian Instruments, Beijing, China).

### 2.9. Statistical Analysis 

All data were represented as the mean ± standard deviation (S.D.). Statistical analysis was performed using an independent t-test or one-way ANOVA test. A *p*-value less than 0.05 (*p* < 0.05) was considered statistically significant.

## 3. Results

### 3.1. The Cytotoxicity of Heavy Metals and Curcumin in HepG2 Cells

**Figure 1:**
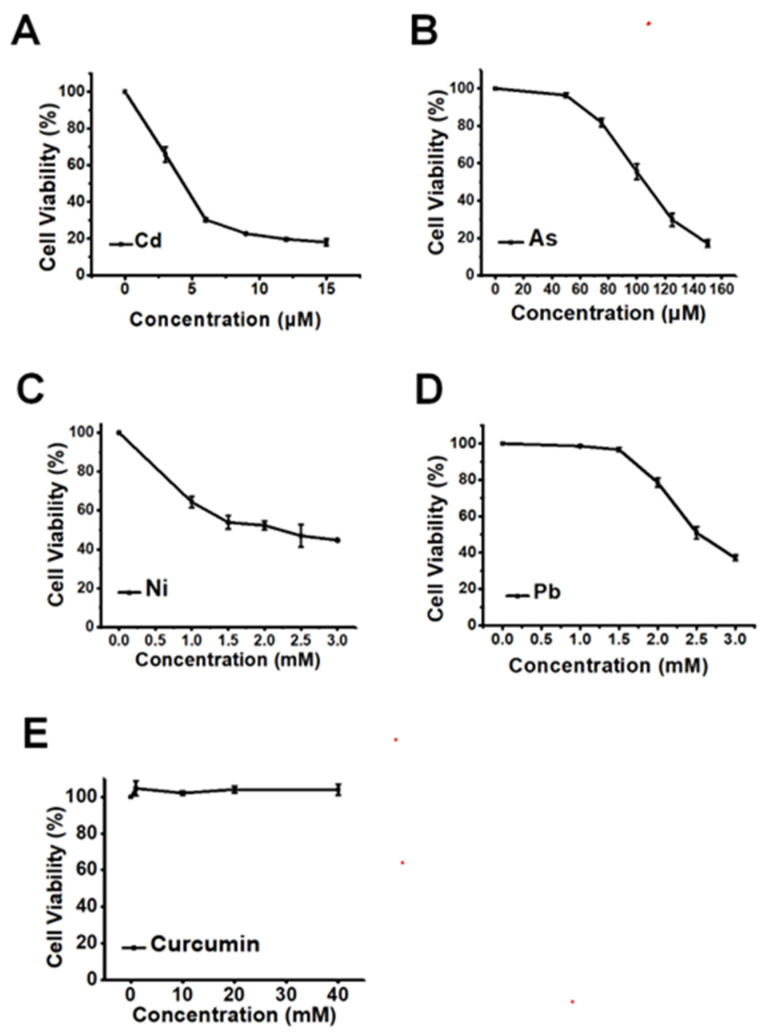
The cytotoxicity of various heavy metals and curcumin in HepG2 cells (*n* = 3). The dose-dependent cellular viabilities decline is induced by (**A**) Cd, (**B**) As, (**C**) Ni and (**D**) Pb. (**E**) The cell viability of HepG2 cells exposed to a serious concentration of curcumin.

The cytotoxicity of heavy metals, including Cd, As, Ni, and Pb, at graded concentrations was first tested in HepG2 cells, to find a comparable concentration for the different types of heavy metals. As shown in Figure 1A–D, the four kinds of heavy metals all induced significant dose-dependent cytotoxicity in HepG2 cells when the concentrations of heavy metals were increased. To compare the antagonistic efficiencies of curcumin against different heavy metals, 6 µM Cd, 100 µM As, 2 mM Ni, and 2.5 mM Pb, which all elicited approximately 50% decline of cellular viabilities, were selected in the following study. In addition, the non-toxic concentration of curcumin should also be considered to avoid the potential toxic response of the antidote itself. As depicted in Figure 1E, no obvious cellular viability reduction could be observed when the concentration of curcumin was lower than 40 μM. Therefore, 5, 10 and 20 μM curcumin were employed for the following detoxification research.

### 3.2. The Antagonistic Effects of Curcumin on the Cytotoxicity of Heavy Metals

**Figure 2:**
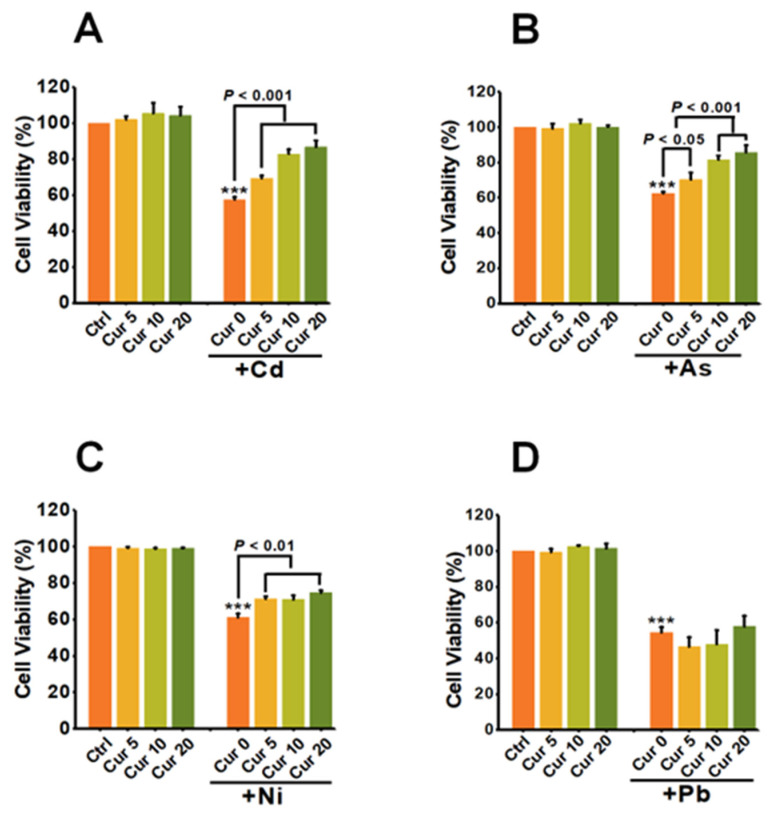
The effects of curcumin on the cytotoxicity of heavy metals (*n* = 3). HepG2 cells were pre-treated with curcumin for 8 h, followed by (**A**) Cd, (**B**) As, (**C**) Ni and (**D**) Pb treatment for another 24 h. The concentrations of curcumin were 5, 10 and 20 μM, respectively. The concentrations of heavy metals were 6 μM for Cd, 100 μM for As, 2 mM for Ni and 2.5 mM for Pb, respectively. *** *p* < 0.001, compared to the untreated group.

As shown in Figure 2A, the cellular viability of Cd at 6 μM was 57.50% ± 1.41 (*p* < 0.001), which was greatly elevated by a pre-treatment with 5, 10 and 20 μM curcumin to 69.33% ± 1.54 (*p* < 0.001), 82.81% ± 2.57 (*p* < 0.001), 86.72% ± 3.61 (*p* < 0.001), respectively. These data indicated a dose-dependent antagonistic effect on Cd-induced cytotoxicity. The maximum detoxification efficiency of curcumin against Cd was 50.82% ± 3.61 (*p* < 0.001) when the concentration of curcumin was 20 μM (Figure 2A). Pre-exposure with curcumin also induced similar dose-dependent antagonistic effects on As- and Ni-induced cytotoxicity, with the maximum antagonistic efficacy of curcumin being 37.42% ± 4.11 (*p* < 0.001) for As and 22.03% ± 5.95 (*p* < 0.01) for Ni (Figure 2B,C). Unlike the above three heavy metals, no obvious antagonistic response could be observed in curcumin and Pb co-treatment cells (Figure 2D).

**Figure 3:**
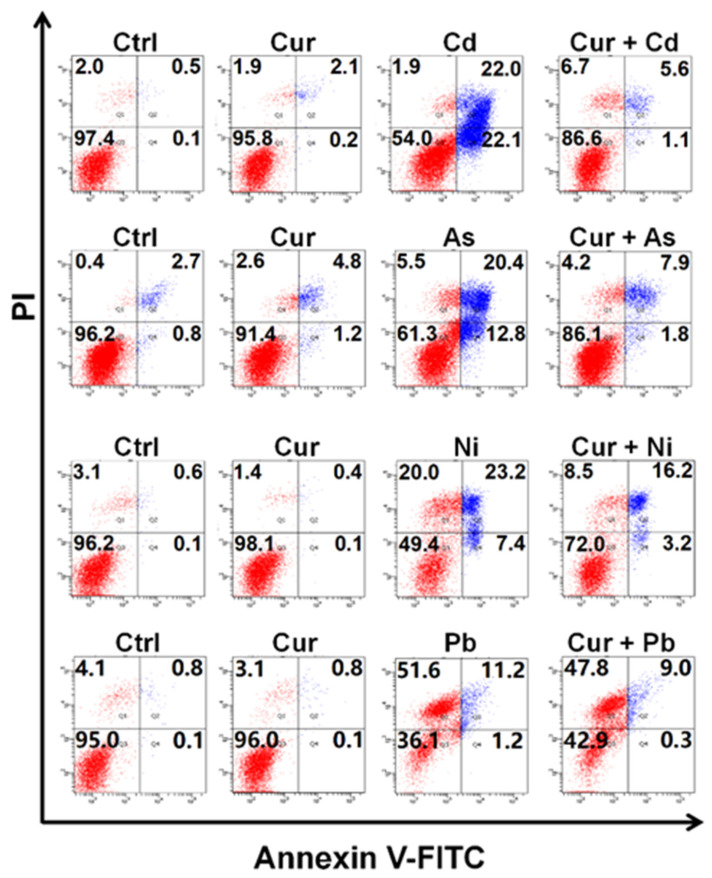
The apoptotic levels of HepG2 cells co-exposed to curcumin and various heavy metals. The concentrations of curcumin and heavy metals were 20 μM for curcumin, 6 μM for Cd, 100 μM for As, 2 mM for Ni and 2.5 mM for Pb, respectively.

Figure 3 indicates the co-effects of curcumin and various heavy metals on apoptosis. The value of 20 μM curcumin used in this study had little effect on inducing apoptosis; in contrast, a significant elevation in apoptotic rates could be detected in Cd-, As- and Ni-treated HepG2 cells. Pre-treatment with curcumin for 8 h sharply relieved the apoptosis elicited by Cd, As and Ni, which itself had little impact on apoptosis induction. The apoptotic rates declined from 44.1% to 6.7%, 33.2% to 9.7%, and 30.6% to 19.4% for Cd, As and Ni, respectively (Figure 3), indicating an obvious antagonistic effect on the cytotoxicity of these three heavy metals. Similar to the observation of the cellular viability assay, scarcely any amelioration could be detected in Pb-exposed cells after curcumin pretreatment (Figure 3). 

**Figure 4:**
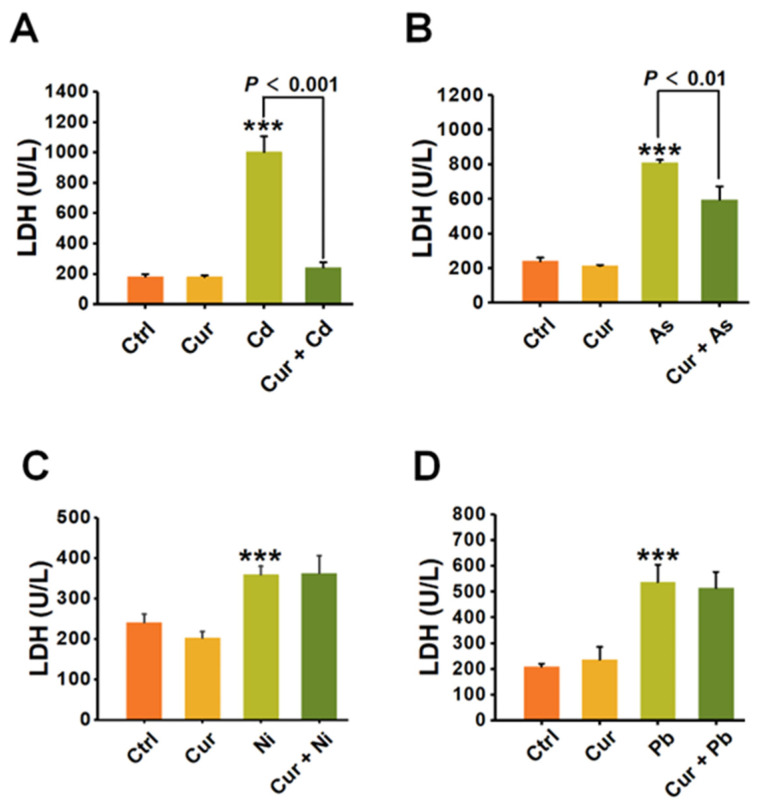
The impacts of curcumin on cell membrane damage induced by heavy metals (*n* = 3). The HepG2 cells are pre-treated with curcumin and incubated with (**A**) Cd, (**B**) As, (**C**) Ni and (**D**) Pb, respectively. The LDH concentrations in the cell supernatants were detected for evaluating the damage to the cell membrane. The concentrations of curcumin and heavy metals were 20 μM for curcumin, 6 μM for Cd, 100 μM for As, 2 mM for Ni and 2.5 mM for Pb, respectively. *** *p* < 0.001, compared to the untreated group.

To further verify the antagonistic effects of curcumin on the cytotoxicity induced by distinct heavy metals, LDH release was analyzed for evaluating the ability of curcumin to restore the membrane damage caused by heavy metals. As depicted in Figure 4, all four types of heavy metals, including Cd, As, Ni, and Pb, significantly destructed the cellular membrane and sharply up-regulated the level of LDH released into the cell culture medium. Pre-treatment with curcumin inhibited the LDH release induced by Cd, As, Ni and Pb from 1003.91 ± 102.87 (*p* < 0.001) to 242.09 ± 33.66 (*p* < 0.001), 810.05 ± 15.32 (*p* < 0.001) to 595.06 ± 76.94 (*p* < 0.01), 359.37 ± 20.53 (*p* < 0.001) to 362.31 ± 43.83, and 537.39 ± 66.25 (*p* < 0.001) to 513.53 ± 63.16, respectively (Figure 4). The antagonistic effects of curcumin on LDH release were 75.89%, 26.54%, 0% and 0.41% for Cd, As, Ni and Pb, separately (Figure 4). 

The above studies, including the cellular viability assay, apoptosis detection and LDH release evaluation, all pointed out the conclusion that distinct antagonistic efficiencies of curcumin exist when combating the toxicity of different heavy metals. Curcumin had the strongest detoxification efficacy on Cd- and As-elicited cytotoxicity, while no effective antagonism could be observed against the cytotoxicity caused by Pb.

### 3.3. The Protective Roles of Curcumin on DNA Damage Caused by Heavy Metals

**Figure 5:**
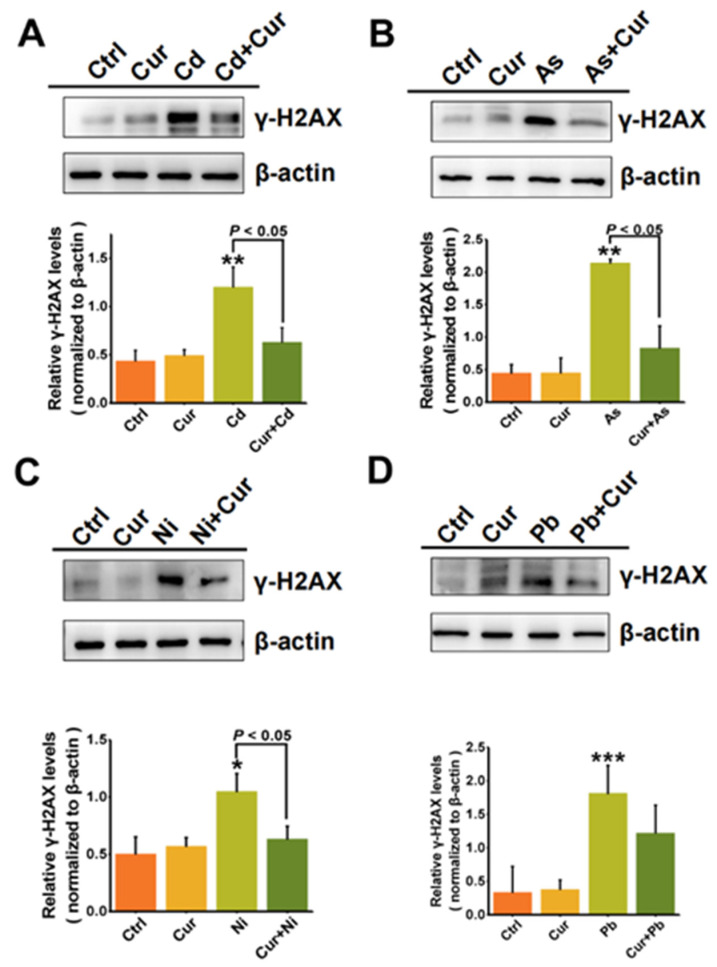
The antagonistic effects of curcumin on the genotoxicity of heavy metals (**A**) Cd, (**B**) As, (**C**) Ni and (**D**) Pb. Western blotting detection of the γ-H2AX protein levels in HepG2 cells exposed to curcumin (10 μM), Cd (6 μM), As (100 μM), Ni (2 mM), Pb (2.5 mM) and their co-treatments. *β*-actin served as a loading control. γ-H2AX levels were quantified by a densitometric analysis relative to β-actin. * *p* < 0.05, ** *p* < 0.01, *** *p* < 0.001.

In order to investigate the detoxification effects of curcumin on the genotoxicity induced by various heavy metals, the generation of phosphorylated histone H2AX (γ-H2AX) was examined, which acts as a major indicator of nuclear DNA double-strand breaks (DSBs) [32]. As indicated by a western blotting assay (Figure 5), significant elevation of γ-H2AX protein expression elicited by the four types of heavy metals could all be remarkably down-regulated via curcumin pre-treatment, which itself had minimal effects on the expression of γ-H2AX. The inhibition efficiencies of curcumin on the γ-H2AX protein expression were 50%, 83%, 40.4%, and 32.6% for Cd, As, Ni and Pb, as quantified by the densitometric analysis in Figure 5. Notably, a more significant antagonistic response of curcumin could be observed for the genotoxicity compared to the cytotoxicity elicited by heavy metals, especially for Pb and Ni. treatments. 

### 3.4. Curcumin Alleviated the Oxidative Stress and the Bioaccumulation of Heavy Metals

**Figure 6:**
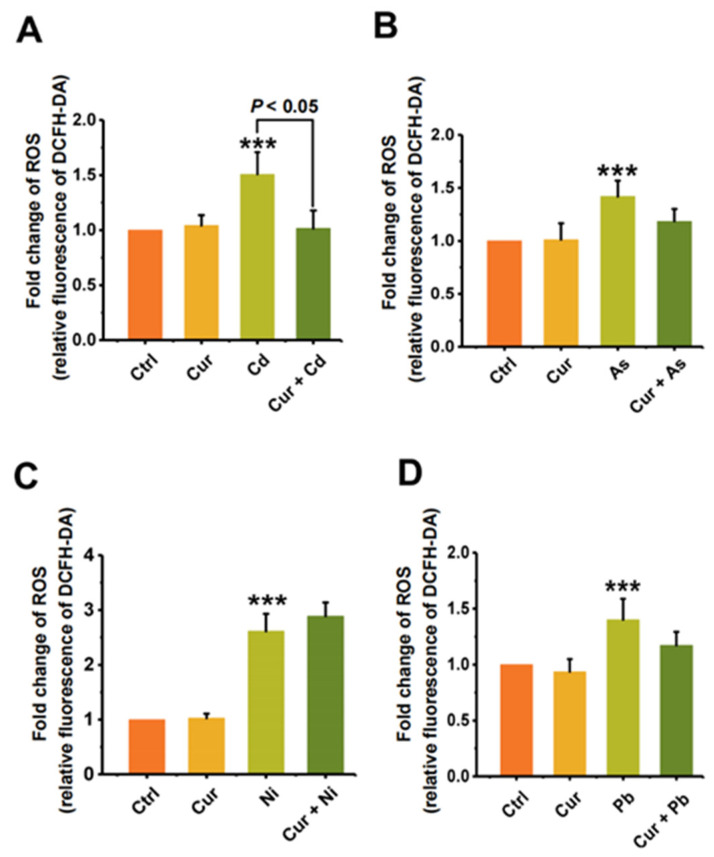
The protective effect of curcumin on the oxidative stress induced by heavy metals (*n* = 3). The ROS production of (**A**) Cd-, (**B**) As-, (**C**) Ni- and (**D**) Pb-treated cells with and without curcumin co-incubation. The concentrations of curcumin and heavy metals were 20 μM for curcumin, 6 μM for Cd, 100 μM for As, 2 mM for Ni and 2.5 mM for Pb, separately. *** *p* < 0.001, compared to the untreated group.

**Figure 7:**
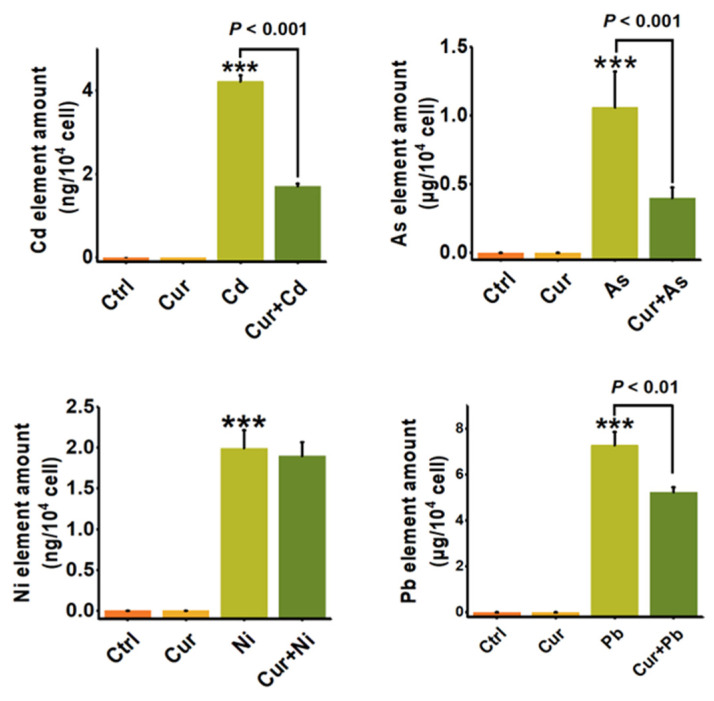
The bioaccumulation of heavy metals in HepG2 cells with or without curcumin (*n* = 3). ICP-MS quantitively detected the cellular amounts of Cd, As, Ni and Pb with and without curcumin pre-treatments. The concentrations of curcumin and heavy metals were 20 μM for curcumin, 6 μM for Cd, 100 μM for As, 2 mM for Ni and 2.5 mM for Pb, separately. *** *p* < 0.001, compared to the untreated group.

Since oxidative stress is one of the important toxic mechanisms of heavy metals, we first elucidated the mechanism of curcumin detoxification from the aspect of its anti-oxidative capacity. As shown in Figure 6, the level of cellular ROS was elevated after stimulation by all four types of heavy metals. Pre-treatment with curcumin inhibited the cellular ROS level induced by Cd, As, Ni, and Pb from 1.51 ± 0.20 (*p* < 0.001) to 1.02 ± 0.16 (*p* < 0.05), 1.42 ± 0.15 (*p* < 0.001) to 1.18 ± 0.12, 2.49 ± 0.32 (*p* < 0.001) to 2.75 ± 0.25, and 1.40 ± 0.19 (*p* < 0.001) to 1.17 ± 0.12, respectively (Figure 6). The inhibition efficiencies of curcumin against distinct heavy metals were 32.45%, 16.9%, 16.43%, and 0% for Cd, As, Pb, and Ni, separately. It can be seen from the results in Figure 6 that curcumin showed different antagonistic effects on the cytotoxicity induced by different heavy metals. Apart from anti-oxidation, we further evaluated the bioaccumulation of heavy metals with and without curcumin co-incubation, as metal chelation is also considered to be a vital mechanism for the protection role of curcumin. Similar to the trend of ROS alteration, curcumin exhibits different abilities to inhibit metal ion accumulation; the reduction efficacies of curcumin were 62.51% ± 0.05 (*p* < 0.001), 62.26% ± 0.08 (*p* < 0.001), 28.16% ± 0.17 (*p* < 0.01) and 4.8% ± 0.02 for Cd, As, Pb, and Ni, respectively (Figure 7), indicating that inhibiting the ROS production and bioaccumulation of heavy metals both contributed to the antagonistic role of curcumin against Cd, As and Pb.

## 4. Discussion

Widely distributed heavy metal pollution has become a serious environmental risk to ecosystems and human beings globally. Environmental exposure to heavy metals through ingestion, inhalation, and skin contact has caused severe damage to various organs, including the liver, kidneys, lungs, heart, skin, and even brain [38]. However, few effective approaches can be used to prevent and cure heavy metal poisoning in current clinical treatment. Scattered studies have suggested that curcumin, a natural product of polyphenols, holds great promise in protecting organisms from the harmful effects caused by heavy metals, however, the inherent regularity of curcumin in antagonizing different heavy metals is largely unknown. 

Through systematically comparing the protective role of curcumin against four typical types of heavy metals under the same experimental condition, significant differences were observed. Curcumin exhibited dominant detoxification efficiency against Cd and As, while negligible protection could be detected on the cytotoxicity of Ni and Pb, especially for Pb (Figure 2, Figure 3 and Figure 4). Since metal chelation and anti-oxidation are the main antagonistic mechanisms for curcumin [28,39,40], the discrepant antagonistic capacity possibly originated from the different heavy metal-ligand complexation reactions. Infrared spectrum analysis suggested that the hydroxyl groups and β-diketones structure in curcumin play a vital role in chelating heavy metals [41]. The binding ratios of heavy metals and curcumin were one to one or one to more, depending on the radius of the metal ions [42]. For example, Cd with a relatively small radius binds curcumin at a ratio of 1:1, while the ratio of the Pb-curcumin complex was 1:2 [42]. The binding ratio of 1:1 was proved to be more active than 1:2 in scavenging superoxide anion radicals through proton transfer or electron transfer, thus leading to a better detoxification efficacy of curcumin against the toxicity of Cd than Pb [43].

Genotoxicity has been certified to be a serious safety concern of heavy metals, since various heavy metals, such as Cd, As, Ni, and their compounds, have been classified as Group I carcinogens by IARC. Through assessing the generation of γ-H2AX, a protein marker of DNA double-strand break, our results indicated that curcumin possesses an excellent protective role for genotoxicity elicited by all the four tested heavy metals (Figure 5), while little protection could be observed for the cytotoxicity of Ni and Pb (Figure 2, Figure 3 and Figure 4). The antagonistic capacity of curcumin on genotoxicity was derived from two aspects: on one hand, curcumin has the ability to protect DNA from oxidative damage induced by singlet oxygen [44]; on the other hand, the shape and size of curcumin make it suitable to directly bind to the small groove of DNA which is helpful for resisting DNA damage [45]. Additionally, DNA double-strand breaks are considered to be the most severe damage, which will lead to genomic instability and even cell death if the injury cannot be fully repaired [46,47]. DNA double-strand breaks can be recognized as one of the earlier initial events for cytotoxicity, while cytotoxicity is a complex and final outcome for various cellular harmful effects. Therefore, a western blotting assay demonstrated the more obvious protective role of curcumin on the genotoxicity than cytotoxicity induced by heavy metals. 

Anti-oxidation and metal chelating were considered to be the primary mechanisms responsible for the protective role of curcumin against heavy metal-induced adverse effects [28,39,40], the mechanisms of which were determined by the chemical structure of curcumin. The presence of the phenolic, β-diketone and methoxy groups all contribute to the direct free-radical-scavenging activity of curcumin [48]. The keto-enol functionality and the aromatic ring system are crucial for curcumin to be an activator of Nrf2, which stabilizes Nrf2 by blocking the ubiquitination of Nrf2, and upregulates the expression of a series of anti-oxidative enzymes, including superoxide dismutase (SOD), catalase (CAT) and so on, thus playing an indirect role in antagonizing the oxidative stress elicited by heavy metals [49]. Additionally, the ketone aldehyde group contained in curcumin has strong metal chelating activity, which will then carry the heavy metals excreted from the organisms [28,40]. Through detecting the ROS production (Figure 6) and the cellular accumulation of heavy metals (Figure 7), our results confirmed the conclusion that both anti-oxidation and bioaccumulation reduction contributed to the detoxification ability of curcumin against heavy metals. Similar protective mechanisms of curcumin against the adverse effects of heavy metals were also proved in other studies. Recent studies have demonstrated that curcumin can protect against Cd-induced damage by activating the anti-oxidative signaling pathway of Nrf2/ARE [7,50] Curcumin could reduce the neurotoxicity and tissue damage elicited by Cd and Pb by chelating these heavy metals. [42,51]. Due to its excellent activity of scavenging intracellular ROS activity, curcumin could be used as an effective antioxidant for the protection of polar ROS induced by heavy metals in the cytoplasm [28].

In addition, negligible effect of curcumin could be observed on relieving the ROS production and bioaccumulation of Ni, while curcumin exhibit significant antagonistic role in Ni-elicited genotoxicity (Figure 5C), indicating that other special mechanism should be evolved in the interaction between curcumin and Ni. Recent studies had re-ported that curcumin inhibits Ni-induced invasion and migration by down-regulating TLR4/NF-B signaling pathway, which is an important initiating factor in the inflam-matory cascade reaction in cells [52]. Besides, Brunella Perfetto et al. showed that cur-cumin inhibits Ni-induced skin damage and tumor formation by downregulating the expression of the Matrix metalloproteinases (MMPs) family which can almost degrade various protein components in the extracellular matrix (ECM), destroy the histological barrier of tumor cell invasion, and play a key role in tumor invasion and metastasis [53]. In our experiments, the mechanism involved in antagonizing the genotoxicity of Ni by curcumin are still needed to be further elucidated in the future.

## 5. Conclusions

In summary, although curcumin has been reported to be a broad-spectrum antidote for heavy metals, our present data points out that significant differences in the detoxification ability exist among distinct types of heavy metals. The protective efficacy of curcumin for Cd- and As-induced toxicity was more obvious than for Pb and Ni. Both anti-oxidation and metal chelation contributed to the antagonistic efficiency of curcumin against heavy metals. Considering the remarkable bioactivity and wide application of curcumin in biomedicine, it is worthwhile to further investigate the protective effect of curcumin against many other contaminants and elucidate the underlying mechanism in vitro and in vivo. 

## Data Availability

Not applicable.
